# PARP1 Inhibitors: antitumor drug design

**Published:** 2015

**Authors:** N. V. Malyuchenko, E. Yu. Kotova, O. I. Kulaeva, M. P. Kirpichnikov, V. M. Studitskiy

**Affiliations:** Lomonosov Moscow State University, Leninskie Gory, 1/12, Moscow, 119991, Russia; Fox Chase Cancer Center, Philadelphia, PA, 19111-2497, USA

**Keywords:** PARP1 inhibitors, poly (ADP-ribose) polymerase 1, antitumor agents

## Abstract

The poly (ADP-ribose) polymerase 1 (PARP1) enzyme is one of the promising molecular targets for the discovery of antitumor drugs. PARP1 is a common nuclear protein (1–2 million molecules per cell) serving as a “sensor” for DNA strand breaks. Increased PARP1 expression is sometimes observed in melanomas, breast cancer, lung cancer, and other neoplastic diseases. The PARP1 expression level is a prognostic indicator and is associated with a poor survival prognosis. There is evidence that high PARP1 expression and treatment-resistance of tumors are correlated. PARP1 inhibitors are promising antitumor agents, since they act as chemo- and radiosensitizers in the conventional therapy of malignant tumors. Furthermore, PARP1 inhibitors can be used as independent, effective drugs against tumors with broken DNA repair mechanisms. Currently, third-generation PARP1 inhibitors are being developed, many of which are undergoing Phase II clinical trials. In this review, we focus on the properties and features of the PARP1 inhibitors identified in preclinical and clinical trials. We also describe some problems associated with the application of PARP1 inhibitors. The possibility of developing new PARP1 inhibitors aimed at DNA binding and transcriptional activity rather than the catalytic domain of the protein is discussed.

## INTRODUCTION

Modern drug discovery and design are based on molecular targeting. The poly (ADP-ribose) polymerase 1 (PARP1) enzyme is one of the targets used in anticancer drug design. It is involved in many cellular processes, from DNA repair to cell death [1]. Recently, recognition of DNA breaks by the PARP1 enzyme was demonstrated to be one of the earliest events that occur upon DNA damage. Once DNA strand breaks occur, in particular due to alkylating agents and radiation, PARP1 binds to the break sites using the so-called “zinc fingers” located in the DNA-binding domain of PARP1 and simultaneously synthesizes oligo-(ADP-ribose) or poly-(ADP-ribose) chains, which are covalently bound to various acceptor proteins or the PARP1 molecule, by transferring an ADP-ribose moiety from NAD^+^. This leads to chromatin decondensation at the break site, facilitating access for repair enzymes. Modified poly-(ADP-ribosyl)ated chromatin proteins attract chromatin remodeling factors. One of the key mechanisms of PARP1-dependent decondensation is based on the fact that an activated PARP1 facilitates the removal of the H1 linker histone from transcription initiation sites. Removal of H1 leads to chromatin decondensation, which allows repair enzymes to attack the damaged DNA sites. It should be noted that DNA repair with active involvement of PARP1 occurs only upon minimal genotoxic damage. Stronger damage triggers apoptosis, while more extensive DNA damage results in overactivation of PARP, leading to cell necrosis.

There is abundant data on the involvement of PARP1 in carcinogenesis. Loss of PARP1 leads to disturbances in the DNA repair process and inhibition of the transcription of several genes involved in DNA replication and cell cycle regulation. Underexpression of PARPI leads to genome shuffling and chromosomal abnormalities and may contribute to overall genome instability. At the same time, increased PARP1 expression is observed in melanomas and lung and breast tumors [2-7]. In this case, the increased expression is considered to be a prognostic feature associated with a poor survival prognosis [8]. A high level of PARP1 expression was shown to correlate with a more aggressive phenotype of breast cancers (BCs) (estrogen-negative BC) [9]. PARP1 expression may correlate with tumor resistance to therapy [10]. This higher “malignancy” is apparently due to the fact that the increased PARP1 expression facilitates damaged DNA repair and, thereby, overcoming the genetic instability characteristic of transformed cells.

There are various mechanisms of the pro-tumor activity of PARP1. In some cases, they are mediated by various tumor-associated transcription factors. Carcinogenesis can be caused by PARP1-dependent deregulation of the factors involved in the cell cycle and mitosis, as well as the factors regulating the expression of the genes associated with the initiation and development of tumors [11]. The relationship between PARP1 and the NF-kB factor has been revealed. PARP1 was found to co-regulate the NF-kB activity and lead to increased secretion of pro-metastatic cytokines. The NF-kB signaling cascade is known to be essential for tumor growth [12]. Inhibition of PARP1 disables a proinvasive phenotype [13, 14]. PARP1 is known to control the expression of heat shock protein 70 (HSP70) [15, 16], which significantly contributes to the survival of tumor cells and their resistance to antitumor agents [17]. PARP1 interacts with the p21 protein, which controls the cell cycle. This may also promote a tumor phenotype [18]. The p21 protein directly interacts with PARP1 during DNA repair, and p21 knockdown leads to an increased enzymatic activity of PARP1. Expression of p21 in tumors is often suppressed due to p53 regulation [19], which may explain the possible role of PARP1 in carcinogenesis. PARP1 was also found to be involved in the hormone-dependent regulation of carcinogenesis. In prostate cancer cells expressing the androgen receptor (AR), PARP1 is recruited to the sites of AR localization and stimulates AR activity [20]. Similar chromatin-dependent mechanisms with the participation of PARP1 are involved in the estrogen-dependent regulation of gene expression in breast cancer (BC).

Since PARP1 is a key enzyme regulating certain carcinogenic changes in the cell, it is regarded as an important molecular target for designed antitumor agents and PARP1 inhibitors are considered to be promising anticancer drugs.

## THE HISTORY OF PARP1 INHIBITOR DESIGN

Since the effect of radiation therapy and many chemotherapeutic approaches to cancer is determined by DNA damage, PARP1 inhibitors can be used to enhance conventional methods and act as chemosensitizers and radiosensitizers. In cells treated with anticancer agents, PARP1 inhibition suppresses the repair of potentially lethal damage and may lead to the destruction of abnormal cells. Similarly, PARP1 inhibitors in some cases increase the efficacy of DNA-alkylating agents (e.g., Temozolomide) and topoisomerase I inhibitors (e.g., topotecan), as well as ionizing radiation. PARP1 inhibitors are also effective in radiosensitization of tumor cells. Along with the synergistic effect of PARP1 inhibitors and other DNA-damaging antineoplastic agents, a direct toxic effect of PAPR1 inhibitors is observed in some tumor cells.

**Fig. 1:**
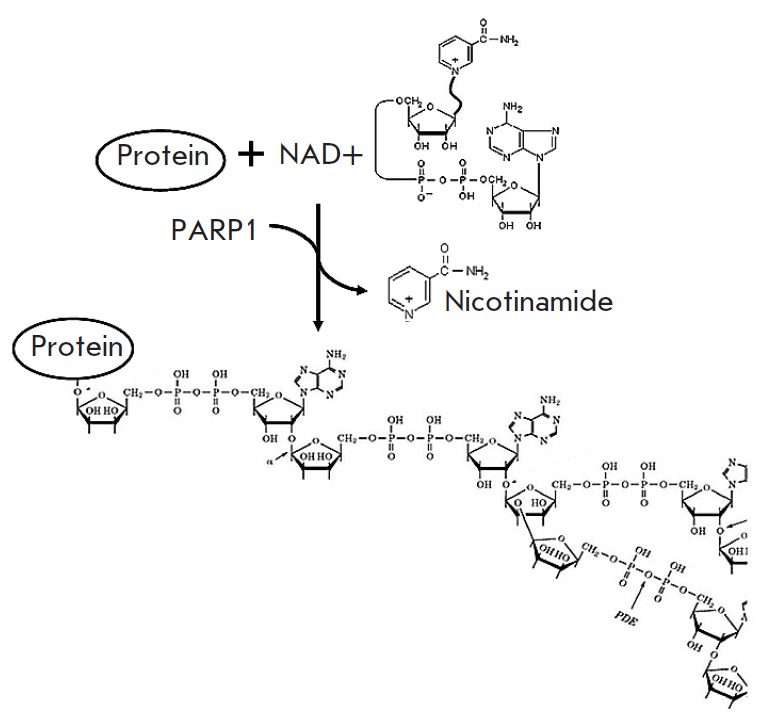
Protein poly (ADP-ribosyl)ation reaction

**Fig. 2:**
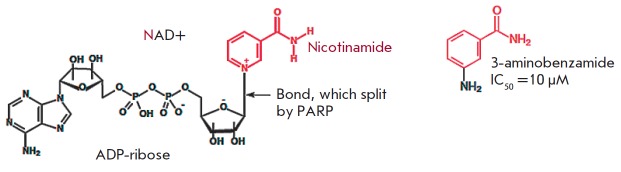
First-generation PARP1 inhibitor, 3-aminobenzamide (3-AB). A nicotinamide pharmacophore group is shown in red

The first generation of typical PARP1 inhibitors, nicotinamide analogues, was developed about 30 years ago based on observations that nicotinamide, a second product of the PARP1-catalyzed reaction, causes moderate inhibition of the reaction (*Fig. 1*). In first-generation PARP1 inhibitors, the heterocyclic nitrogen atom at the third position was replaced by a carbon atom, which led to the development of a class of benzamide analogues [21]. Substitution at the third position led to improved drug solubility (*Fig. 2*). Investigation of the activity of 3-substituted benzamides (e.g., 3-aminobenzamide, 3-AB) provided a better understanding of the PARP1 function. These drugs turned out to have a cytotoxic effect on tumor cells when used concomitantly with genotoxic stress agents [22]. Despite the encouraging results in the investigation of first-generation PARP1 inhibitors, benzamides proved ineffective in practice. In preclinical trials in cell cultures, they had to be used at millimolar concentrations, which made them inappropriate for trials in animals. Furthermore, benzamides inhibited other cellular pathways [23]. Nevertheless, they provided the basis for developing more effective drugs. Virtually all currently used PARP1 inhibitors comprise the nicotinamide/benzamide pharmacophore group.

**Fig. 3:**
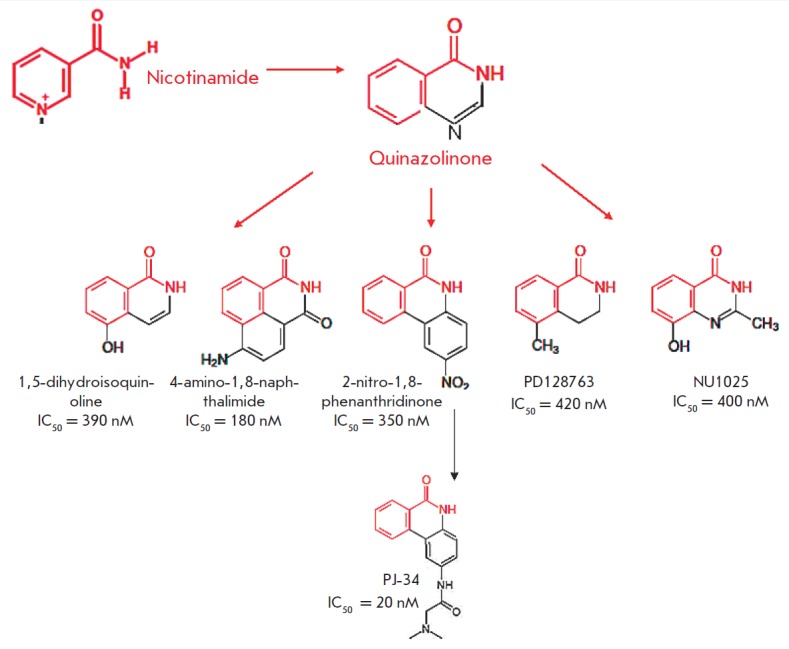
Second-generation inhibitors. A nicotinamide pharmacophore group is shown in red

In the 1990s, more effective second-generation PARP1 inhibitors were developed based on quinazoline analogues (in particular, 1,5-dihydroisoquinoline). This group of compounds includes isoquinolines, quinazolinediones, phthalazinones, and phenanthridinones. Second- generation PARP1 inhibitors were more effective and target-specific [24]. Some of these compounds became the basis for further development of various drug groups (*Fig. 3*). In particular, the production of phenanthridinones led to the development of PJ-34, which was further used in clinical trials (CTs) [25]. An alternative approach (chemical synthesis based on the analysis of the structure and activity relationship, SAR) led to the identification of 3,4-dihydro-5-methyl-1-[2H]-isoquinolinone (PD128763) and 8-hydroxy-2-methylquinazolin- 4-[3H]-one (NU1025). Both of these compounds are ~50 times more effective PARP1 inhibitors than 3-AB.

Later on, more potent inhibitors were developed on the analogy with existing ones. All of them contained a carboxamide group of the benzamide pharmacophore in the second aromatic ring. This was the modification that proved crucial for increasing the activity of inhibitors. The reasons explaining the relationship between these structural features and the increased activity became apparent after structural studies. Crystallization of PARP1 inhibitors showed that the carboxamide group forms several important hydrogen bonds with Ser904-OG and Gly863-N in the catalytic domain of PARP1, which improves the interaction between the heterocycle of these inhibitors and the protein [26]. In this case, the amide group of more effective inhibitors (PD128763, 4ANI, and NU1025) is restricted in the het erocyclic ring. The significance of aromatic (π-π) interactions between a phenolic group of PARP1 inhibitors and a phenolic group of Tyr907 of the PARP1 protein was also revealed. On the basis of the structural analysis of NU1085 binding, several tricyclic lactam indoles and benzimidazoles were developed in which a carboxamide group was introduced in a favorable orientation by its inclusion into a 7-membered ring [27-30]. These compounds (for example, AG14361) are capable of forming crucial hydrogen bonds with Gly863, Ser904, and Glu988 of the PARP1 protein [31].

**Fig. 4:**
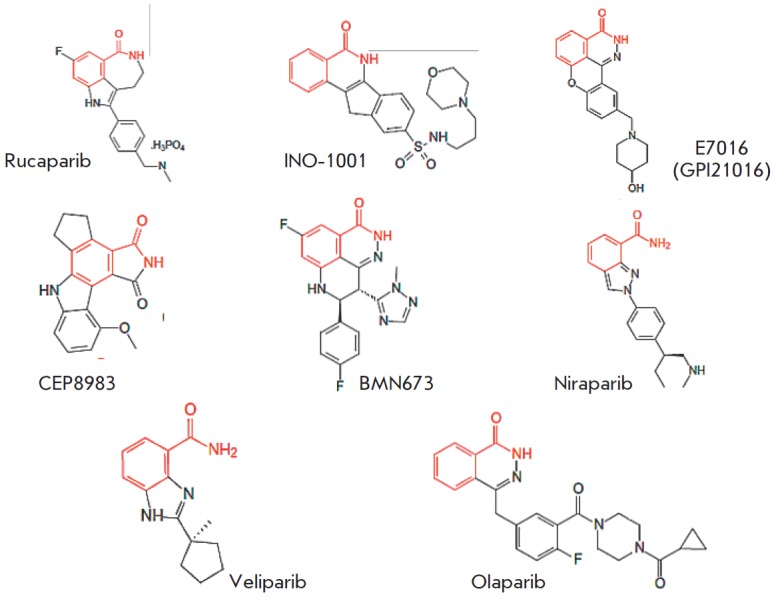
Structures of third-generation PARP1 inhibitors. A nicotinamide pharmacophore group is shown in red

Further search led to the development of more potent third-generation PARP inhibitors, the first characterized representative of which was rucaparib (*K*i = 1.4 nM) [32]. At present, a number of benzimidazole- based PAPP1 inhibitors of the third generation have been synthesized. Many of them (e.g., rucaparib, iniparib, olaparib, veliparib, niraparib, talazoparib, CEP-9722, and E7016) are currently undergoing clinical trials (see reviews [33-38], *Fig. 4*, *Table*).

**Table:** Clinical trials of PARP1 inhibitors. Data were borrowed from reviews [42, 43]

Name	Therapy	Tumors	CT phase
Rucaparib AG014699	Monotherapy	BRCA mutant lung cancer, ovarian cancer	2
Rucaparib	+temozolomide	Solid tumors, melanoma	2
Rucaparib	+carboplatin	Solid tumors	1
Olaparib	Monotherapy	Solid tumors, BRCA, TNBC/HGSOC carriers	2
Olaparib	+topotecan	Solid tumors	1
Olaparib	+dacarbazine	Solid tumors	1
Olaparib	+bevacizumab	Solid tumors	1
Olaparib	+paclitaxel	Ovarian Cancer	2
Olaparib	+paclitaxel	Stomach cancer	2
Olaparib	+cisplatin	Solid tumors	1
Veliparib ABT-888	Monotherapy	Solid tumors	1
Veliparib	+topotecan	Solid tumors	1
Veliparib	+carboplatin	Solid tumors	1
Veliparib	+temozolomide	Solid tumors, liver tumors, prostate cancer	2
Veliparib	+cyclophosphamide	Solid tumors and lymphomas	2
INO-1001	+temozolomide	Melanoma	1
MK4827	Monotherapy	Solid tumors and lymphoma	2
MK4827	+temozolomide	Ovarian cancer/glioblastoma	1
MK4827	+doxorubicin	Ovarian cancer/glioblastoma	1
CEP-9722	Monotherapy	Solid tumors	1
CEP-9722	+temozolomide	Lymphomas	1
BMN-673	Monotherapy	Solid tumors	1
Iniparib (BSI-201)	+gemcitabine +carboplatin	mTNBC	2
Iniparib	+gemcitabine +cisplatin	Lung cancer	2
Iniparib	+gemcitabine +carboplatin	mTNBC	3

## MECHANISMS OF ACTION OF PARP1 INHIBITORS: DIRECT ANTITUMOR ACTION

PARP1 inhibition leads to failure of DNA repair. PARP1 is known to bind to single-strand and double-strand DNA breaks in response to DNA damage [39]. In the absence of damage, the PARP1 activity is minimal. However, the appearance of damages causes its immediate and significant (up to 500 times) activation. PARP1 finds DNA breaks, acting as a sensor and providing a rapid recruitment of repair proteins to the break site. PARP1 controls several DNA repair pathways, including base excision repair (BER), nucleotide excision repair (NER), mismatch repair (MMR), and repair of double-strand breaks through homologous recombination (HR) and non-homologous end-joining (NHEJ) [39].

**Fig. 5:**
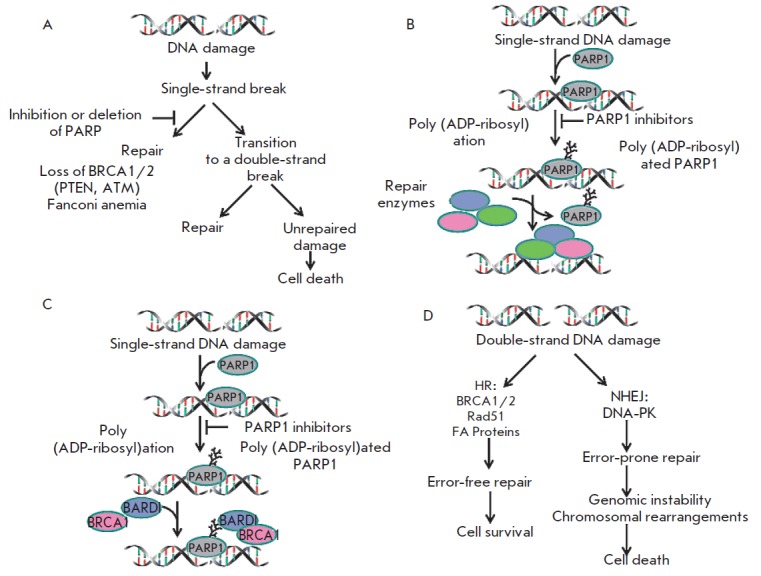
Direct cytotoxic effect of PARP1 inhibitors. *A ***– **inhibition of PARP1 leads to inactivation of a repair system and preservation of spontaneously occurring single-strand breaks (SSBs), which causes formation of double-strand breaks.* B ***– **because of the action of PARP1 inhibitors, PARP1 remains bound to damaged DNA and, thus, cannot dissociate from it and clear the area for PARP1-dependent repair enzymes. *C ***– **in the presence of PARP inhibitors, mutant BRCA1 is less accumulated at the DNA damage site, *D ***– **when double-strand breaks occur in HR-deficient cells, another NHEJ system is activated. As a result, repair errors occur that can lead to genomic instability and cell death

Inhibition of PARP leads to inactivation of the repair system and retention of spontaneous single-strand breaks (SSBs) *(Fig. 5A), *which may induce the subsequent formation of double-strand DNA breaks (DSBs). DSBs can be repaired in two ways, either by “error-free DNA repair” using HR, or by repair with possible replacement of the nucleotides in a sequence by NHEJ [40, 41]. In some tumor cells with disruption in the homologous recombination system (e.g., BRCA-mutated cells), the NHEJ system can be turned on. However, the use of NHEJ in these tumors leads to destabilization of the genome and, eventually, cell death due to rapid accumulation of genetic errors [42-44].

In 2005, there was a breakthrough in the research of PARR1 inhibitors. Two independent groups of researchers demonstrated that BRCA1- and BRCA2-deficient cell lines are sensitive to the direct action of PARP inhibitors. It was the first evidence that PARP1 inhibitors can act as independent remedies in the case of tumors in which certain DNA repair pathways are disrupted [45, 46]. The tumor-associated *BRCA1* gene is known to play an important role in the repair of double-strand breaks through the HR mechanism. BRCA1-deficient cells are characterized by less effective HR, and DNA repair in these cells mainly occurs via the BER system. BRCA2 interacts with the RAD51 protein and also plays a significant role in HR. Cells with mutations in the BRCA2 region responsible for binding to RAD51 exhibit hypersensitivity to DNA damage and chromosomal instability [47]. For example, 10–15% of serious ovarian cancers are hereditary and caused by a mutation in the *BRCA1 *or *BRCA2 *gene*.* HR repair defects arising from mutations in *RAD51, DSS1*, *RPA1, *or *CHK1 *were shown to cause increased sensitivity of cells to PARP1 inhibition [48]. In the case of homologous recombination deficiency, inhibition of DNA damage repair leads to cell death due to the inability to fix all DNA damage.

The direct action of PARP1 inhibitors on tumor cells may also be explained by another mechanism. Because of inhibitor action, PARP1 is believed to remain bound to damaged DNA, and, therefore, it cannot dissociate from the DNA and “clear” the area for PARP1-dependent repair enzymes (*Fig. 5B*).

The third model of the direct action of PARP1 inhibitors is based on the observations of Li and Yu [49], who showed that mutant BRCA1 is less accumulated at the DNA damage site in the presence of PARP1 inhibitors (*Fig. 5C*).

A fourth model of the direct action of PARP1 inhibitors (*Fig. 5D*) was also proposed. According to this model, double-strand breaks in HR-deficient cells result in activation of another NHEJ system [44]. As previously shown, key proteins in this system (Ku70, Ku80, and DNA-PKcs) have PARP1-binding motifs and can be controlled via ADP-ribosylation [50, 51].

In clinical studies, olaparib monotherapy resulted in inhibition of tumors with mutations in *BRCA1 *or* BRCA2 *(breast cancer and ovarian cancer) [52, 53]. In this case, BRCA1- or BRCA2-deficient cells were 57 or 133 times more sensitive to PARP1 inhibition, respectively [46]. However, the efficacy of this therapy was low; a positive response was observed in less than 50% of patients [54]. Therefore, it is very important to correctly identify the prognostic markers of PARP1 inhibitor therapy. Mutations in the *RAD51, NBS1, ATM, ATR, Chk1, Chk2, Rad54, FANCD2, FANCA, 53BP1*,* PALB2, FANCC, *and *PTEN *genes may serve these markers [39, 55-59].

Terminal mutations of the *BRCA1 *or *BRCA2 *gene in tumor cells lead to defects in the homologous DNA recombination system whose normal activity involves both BRCA proteins. In this case, tumor cells become extremely dependent on one of the five other repair systems, with PARP1 being involved in each of them. In the case of homologous recombination deficiency, PARP1 inhibition leads to cell apoptosis because of the impossibility to repair all DNA damage. This process is called “synthetic lethality.” Several studies have shown that administration of PARP1 inhibitors is a promising treatment in patients with tumors arising from defects in the *BRCA *genes*.*

## MECHANISMS OF ACTION OF PARP1 INHIBITORS: SYNERGISTIC ACTION

PARP1 inhibitors do not always have a direct cytotoxic effect on tumor cells. In these cases, the desired effect can be achieved by concomitant administration of PARP1 inhibitors and other DNA-damaging drugs.

## SYNERGISTIC ACTION OF PARP1 INHIBITORS AND DNA METHYLATING AGENTS

As early as in the 1980s, it was shown by the example of 3-AB that PARP inhibitors enhance the action of DNA-methylating agents [22]. DNA-methylating agents, such as dacarbazine (DTIC) and temozolomide (TMZ), are currently widely used in the treatment of brain tumors and melanomas. These drugs are capable of methylating DNA at the O6 and N7 positions of guanine and the N3.position of adenine. Removal of N-methylpurines (N7-MEG and N3-MEA) leads to the emergence of SSBs, while inhibition of PARP1 inactivates repair of this damage [60]. Early studies demonstrated that PD128763 and NU1025 enhance TMZ-induced DNA damage and increase TMZ cytotoxicity 4–7 times when used at lower TMZ concentrations (50–100 times) [61]. Improved efficacy of TMZ (up to 6 times) in the presence of NU1085 was observed in 12 different human tumor lines, independent of their tissue origin and p53 status [62]. A series of benzimidazoles and tricyclic lactam indoles, including AG14361 at a concentration as low as 0.4 μM, enhances TMZ-induced inhibition of LoVo (human colon cancer) cell growth by a factor of 5.3 [30]. This synergistic action of inhibitors of PARP and Topo I was observed in numerous studies *in vitro*. It should be emphasized that PARP1 inhibitors were found to increase the cytotoxicity of TMZ primarily in the S-phase, which is indicative of the synergistic action mechanism. The inhibitors are most likely to cause accumulation of DSBs during replication [63, 64]. An enhanced antitumor activity of TMZ in the presence of various PARP inhibitors* in vivo *was demonstrated in many experiments. Here are some examples. Combined treatment with NU1025 and TMZ increases the survival rate of mice with brain lymphomas [65]. The GPI 15427 inhibitor enhances the TMZ-induced inhibition of tumor growth and the antimetastatic activity in a B16 melanoma model [66]. Veliparib enhances the activity of TMZ in subcutaneous, orthotropous, and metastatic models of human xenografts, including lymphomas and ovarian, lung, pancreatic, breast, and prostate cancers [67]. Interestingly, both GPI 15427 and veliparib pass through the blood-brain barrier and enhance the antitumor activity of TMZ in mice with intracranial melanomas, gliomas, and lymphomas [68]. In children tumor models, rucaparib enhances the antitumor activity of TMZ in neuroblastoma and medulloblastoma xenografts [69]. Complete tumor regression caused by treatment with TMZ and CEP-6800 was observed in mice bearing xenografts U251MG (human glioblastoma) [70] and SW620 (human colon cancer) [32, 71]. These and other data obtained in experiments *in vivo *gave rise to clinical trials of PARP inhibitors together with DNA methylating agents *(see table)*.

## SYNERGISTIC ACTION OF INHIBITORS OF PARP1 AND TOPOISOMERASE I (TOPO I)

Topo I activity is known to be enhanced in some tumors [72]. Topo I inhibitors are used against various forms of tumors. For example, topotecan is used in the treatment of small-cell lung cancer, ovarian cancer, and cervical cancer. Irinotecan is used in the treatment of colon cancer. Topo I introduces temporary damage to DNA to remove the stress accumulated in the DNA during transcription and replication. Topo I inhibitors, e.g., camptothecins, stabilize the Topo I-DNA cleavage complex at a stage where DNA breaks occur. Repair of Topo I-induced damage involves BER/SSB. In this case, cells lacking the key BER protein, XRCC1, are hypersensitive to camptothecin. PARP enzymes are believed to be involved in this process, recruiting XRCC1 to Topo I-dependent DNA breaks [73], which, in turn, recruit tyrosyl-DNA-phosphodiesterase (TDP 1), which removes Topo I from DNA [74]. Furthermore, PARP1 is capable of interacting with Topo I and repairing Topo I-dependent SSBs [75]. Several studies have demonstrated the potentiation of topoisomerase I inhibitors in the presence of PARP inhibitors [30, 32, 71]. Here are some examples. In 1987, Mattern M.R. *et al*. were the first to use PARP inhibitors as potential enhancers of Topo I inhibitors. They showed that 3-AB increases the cytotoxicity of Camptothecin in L1210 cells [76]. Later on, the synergistic action of Topo I and PARP1 inhibitors was extensively studied. It was shown in 12 human tumor cell lines that NU1025 and NU1085 enhance the cytotoxicity of topotecan, regardless of the tissue origin of these lines and p53 status [62]. CEP-6800 and GPI 15427 enhance the chemosensitivity of colon cancer cell lines to Topo I inhibitors [70, 77]. Encouraging results were also obtained in *in vivo *experiments studying the combined effect of PARP and Topo I inhibitors. CEP-6800 increased the irinotecan-dependent inhibition of tumors in mice bearing HT29 xenografts by 60% [70], while olaparib increased the toxicity of topotecan, so that its dose could be reduced by a factor of 8 [78]. These and other results of *in vivo *experiments gave rise to clinical trials of a combined application of PARP inhibitors and Topo I inhibitors *(Table)*.

## SYNERGISTIC ACTION OF PARP1 INHIBITORS AND RADIOTHERAPY

Ionizing radiation causes various damage to DNA, modification of bases, SSBs, and DSBs; the latter are believed to be the most cytotoxic ones. Sensitization of cells which have been treated with PARP inhibitors to ionizing radiation is less significant than their sensitization to chemical compounds and typically increases the cytotoxicity by less than two times. However, given the large number of patients subjected to radiation therapy, this combination may be reasonable. Early studies demonstrated that inhibition of PARP leads to radiosensitization of mammalian cells [79]. Later on, it was shown that various PARP inhibitors (ANI, NU1025, olaparib, and E7016) enhance the radiosensitization efficacy in various cell lines by a factor of 1.3–1.7 [80]. In some studies, PARP inhibitors selectively induced radiosensitization of actively replicating cells in the S-phase [24]. This suggested a mechanism by which PARP inhibition increases the sensitivity to ionizing radiation. The inhibition prevents the repair of SSBs, converting them into DSBs during the movement of the replication fork in the S-phase [81]. This hypothesis is supported by the observation that PARP inhibition leads to the formation of additional γH2AX and RAD51 foci (which is indicative of an increased frequency of homologous recombination repair (HRR) at the stalled replication fork). The ability of cells to recover after potentially lethal damage (PLD) is a predisposing factor for radiation resistance *in vivo*. However, there is a chance for preservation of radioresistant tumor cells that can reproduce the tumor after radiation therapy [82]. PARP1 inhibitors (e.g., PD128763, NU1025, and AG14361) were shown to prevent recovery of tumor cells after PLD [63]. A number of studies have revealed the effectiveness of radiosensitization by PARP1 inhibitors *in vivo*. The PD128763 inhibitor induced a threefold increase in the therapeutic activity of X-rays in mice bearing SCC7, RIF-1, and KHT sarcomas [83]. Preclinical studies demonstrated that veliparib significantly enhances the antitumor activity of ionizing radiation in xenograft models of human colon, lung, and prostate cancers [68, 84, 85].

## PARP1 INHIBITOR EFFECT IN COMBINATION WITH OTHER CYTOTOXIC DRUGS

There is some evidence of the ability of PARP inhibitors to enhance the effect of other antitumor cytotoxins. For example, 6(5H)-phenanthridinone enhances the cytotoxicity of carmustine in mice lymphoma [86]. PJ34 increases the cytotoxicity of doxorubicin in HeLa cells, presumably due to an increased level of topoisomerase II [87]. A similar compound, INO-1001, enhances the antitumor activity of doxorubicin in xenografts of MDA-MB-231 and MCA-K lung cancer cells [88]. Reports of the synergistic action of PARP inhibitors and platinum compounds, such as cisplatin and carboplatin, are contradictory. Nevertheless, some studies demonstrated that PARP1 is activated by cisplatin-induced DNA damage [89], which gave rise to clinical trials of PARP inhibitors combined with cisplatin derivatives*(Table)*.

## APPLICATION ISSUES OF EXISTING PARP1 INHIBITORS AND PROSPECTS FOR NEW INHIBITOR DISCOVERY

Almost all existing PARP1 inhibitors are nicotinamide mimetics, i.e. aimed at binding to the catalytic domain of PARP1 and competition with NAD^+^. In experiments* in vitro, *as well as in a variety of preclinical and some clinical trials, PARP1 inhibitors showed quite good results as antitumor agents. However, a number of problems were uncovered in more systematic, controlled, extensive clinical trials of PARP1 inhibitors. First, compounds inhibiting NAD^+^ binding have a rather low specificity for PARP1 and also block other enzymatic pathways involving NAD^+^. It should be noted that NAD^+^ is a cofactor that interacts with many enzymes involved in a number of cellular processes, and, therefore, competition with NAD^+^ leads to high toxicity. Second, enzymatic PARP1 inhibitors activate viral replication and are contraindicated for patients infected with viruses such as the human T-cell lymphotropic virus (HTLV) or Kaposi’s sarcoma-associated herpes virus (KSHV) [90-92]. Third, the safety issue in long-term administration of existing PARP1 inhibitors still remains open. Tumor cells are known to be able to rapidly acquire resistance to drugs used as a long-term monotherapy [93]. For these reasons, many PARP1 inhibitors did not pass long-term systematic clinical trials. Trials of some PARP1 inhibitors were discontinued as early as at stages I and II due to high toxicity and some side effects. The history of iniparib (BSI-201) is illustrative in this respect. This drug was the most developed compared to the other PARP1 inhibitors and entered a phase III randomized clinical trial.

**Fig. 6:**
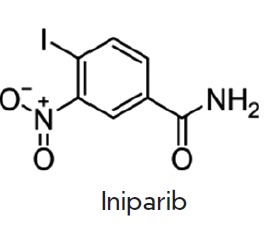
The structure of iniparib

Phase III clinical trials of BSI-201 (iniparib) began in July 2009 to assess the efficacy of this drug in combination with chemotherapy in female patients with metastatic triple-negative breast cancer (mTNBC). The study involved 519 females with mTNBC from 109 centers in the USA. And as early as in 2013, Sanofi- aventis announced the termination of clinical trials as no improvement in patients’ condition and overall survival of patients treated with iniparib and chemotherapy was observed compared to the control group (chemotherapy alone). A number of circumstances led to the failure of clinical trials of iniparib. The main cause for the failure was that preclinical experiments were not complete by the time of group recruitment for clinical trials; very little information on the iniparib action mechanism was gained. Iniparib had been admitted to phase I CTs before the results of preclinical studies were obtained [94, 95]. In this regard, one more fact is interesting: Bipar company, which designed iniparib and the project for Sanofi, did not disclose the compound structure for patent reasons. Later on, it occurred that, unlike all the other PARP1 inhibitors having a similar structure, only iniparib had a flexible carboxyl group capable of rotating around the amide bond, which significantly weakened binding of the inhibitor to PARP1 (*Fig. 6*). One of Sanofi's experts confided that “If Bipar had provided us with the iniparib structure; we would probably have been able to assume that it would not be a good PARP1 inhibitor.” However, despite an insufficient description of the drug (known structure and pharmacodynamic data), the company included it in clinical trials, which cost Sanofi-aventis 285 million dollars.

**Fig. 7:**
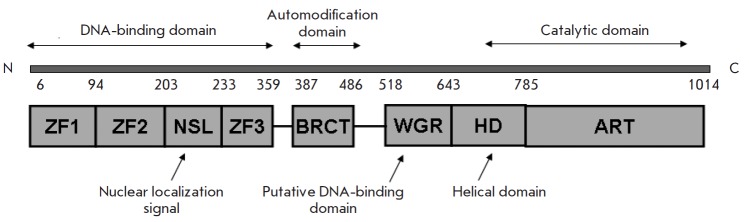
Structural and functional organization of PARP1. The PARP1 structure is composed of three main functional domains: N-terminal DNA-binding domain, internal automodification domain, and C-terminal catalytic domain [108, 109], as well as additional functional sites

The quite high toxicity and some side effects caused by enzymatic PARP1 inhibitors in CTs necessitate alternation in the strategy for new PARP1 inhibitor development. Since PARR1 consists of several functional domains and exhibits accessory, along with enzymatic, activities, in particular DNA-binding and transcriptional ones (*Fig. 7*), PARP1 activity can be regulated by inhibiting these functional domains. In particular, drugs aimed at inhibiting PARP1 binding to DNA are being developed [96]. According to these authors, discovery of compounds capable of preventing PARP1 involvement in the transcription process may lead to the development of a new class of drugs with higher specificity and less severe side effects. More information about the role of PARP1 in transcriptional regulation can be found in [97-101]. The use of a transcriptional system, which was previously obtained by these authors, in mononucleosome and polynucleosome systems enables the discovery and verification of transcriptional inhibitors of PARP1.

In conclusion, it should be noted that PARP1 inhibitors are of great interest and practical value not only in oncology, but also in the treatment of various inflammatory processes, cardiovascular and neurological diseases, as well as age-related diseases. The therapeutic effect of PARP inhibitors in these processes was beyond the scope of this review (see reviews [102-107] for details).

## Abbreviations

PARP1: poly (ADP-ribose) polymerase 1
BER: base excision repair
NER: nucleotide excision repair
MMR: mismatch repair
HR: homologous recombination
NHEJ: non-homologous end joining
SSB: single-strand break
DSB: double-strand break
TMZ: temozolomide
Topo I: topoisomerase 1
CT: clinical trial
PLD: potentially lethal damage

## References

[1] Kraus W.L., Hottiger M.O. (2013). Mol. Aspects Med..

[2] Rodríguez M.I., Peralta-Leal A., O’Valle F., Rodriguez-Vargas J.M., Gonzalez-Flores A., Majuelos-Melguizo J., López L., Serrano S., de Herreros A.G., Rodríguez-Manzaneque J.C. (2013). PLoS Genet..

[3] Nowsheen S., Cooper T., Stanley J.A., Yang E.S. (2012). PLoS One..

[4] Galia A., Calogero A.E., Condorelli R., Fraggetta F., La Corte A., Ridolfo F., Bosco P., Castiglione R., Salemi M. (2012). Eur. J. Histochem..

[5] Csete B., Lengyel Z., Kádár Z., Battyáni Z. (2009). Pathol. Oncol. Res..

[6] Telli M.L., Ford J.M. (2010). Clin. Breast Cancer..

[7] Shimizu S., Nomura F., Tomonaga T., Sunaga M., Noda M., Ebara M., Saisho H. (2004). Oncol. Rep..

[8] Rojo F., García-Parra J., Zazo S., Tusquets I., Ferrer-Lozano J., Menendez S., Eroles P., Chamizo C., Servitja S., Ramírez-Merino N. (2012). Ann. Oncol..

[9] Domagala P., Huzarski T., Lubinski J., Gugala K., Domagala W. (2011). Breast Cancer Res. Treat..

[10] Michels J., Vitale I., Galluzzi L., Adam J., Olaussen K.A., Kepp O., Senovilla L., Talhaoui I., Guegan J., Enot D.P. (2013). Cancer Research.

[11] Simbulan-Rosenthal C.M., Ly D.H., Rosenthal D.S., Konopka G., Luo R., Wang Z.Q., Schultz P.G., Smulson M.E. (2000). Proc. Natl. Acad. Sci. USA..

[12] Dajee M., Lazarov M., Zhang J.Y., Cai T., Green C.L., Russell A.J., Marinkovich M.P., Tao S., Lin Q., Kubo Y., Khavari P.A.. (2003). Nature.

[13] Martín-Oliva D., O’Valle F., Muñoz-Gámez J.A., Valenzuela M.T., Nuñez M.I., Aguilar M., Ruiz de Almodóvar J.M., Garcia del Moral R., Oliver F.J. (2004). Oncogene..

[14] Ohanna M., Giuliano S., Bonet C., Imbert V., Hofman V., Zangari J., Bille K., Robert C., Bressac-de Paillerets B., Hofman P. (2011). Genes Dev..

[15] Tulin A., Spradling A. (2003). Science..

[16] Petesch S.J., Lis J.T. (2008). Cell..

[17] Leu J.I., Pimkina J., Frank A., Murphy M.E., George D.L. (2009). Molecular Cell.

[18] Cazzalini O., Donà F., Savio M., Tillhon M., Maccario C., Perucca P., Stivala L.A., Scovassi A.I., Prosperi E. (2010). DNA Repair..

[19] Abbas T., Dutta A. (2009). Nat. Rev. Cancer..

[20] Schiewer M.J., Goodwin J.F., Han S., Brenner J.C., Augello M.A., Dean J.L., Liu F., Planck J.L., Ravindranathan P., Chinnaiyan A.M. (2012). Cancer Discov..

[21] Purnell M.R., Whish W.J. (1980). Biochem. J..

[22] Durkacz B.W., Omidiji O., Gray D.A., Shall S. (1980). Nature.

[23] Milam K.M., Cleaver J.E. (1984). Science..

[24] Banasik M., Komura H., Shimoyama M., Ueda K. (1992). J. Biol. Chem..

[25] Jagtap P., Szabó C. (2005). Nat. Rev. Drug Discov..

[26] Ruf A., de Murcia G., Schulz G.E. (1998). Biochemistry..

[27] Canan Koch S.S., Thoresen L.H., Tikhe J.G., Maegley K.A., Almassy R.J., Li J., Yu X.H., Zook S.E., Kumpf R.A., Zhang C. (2002). J. Med. Chem..

[28] Skalitzky D.J., Marakovits J.T., Maegley K.A., Ekker A., Yu X.H., Hostomsky Z., Webber S.E., Eastman B.W., Almassy R., Li J. (2003). J. Med. Chem..

[29] Tikhe J.G., Webber S.E., Hostomsky Z., Maegley K.A., Ekkers A., Li J., Yu X.H., Almassy R.J., Kumpf R.A., Boritzki T.J. (2004). J. Med. Chem..

[30] Calabrese C.R., Batey M.A., Thomas H.D., Durkacz B.W., Wang L.Z., Kyle S., Skalitzky D., Li J., Zhang C., Boritzki T. (2003). Clin. Cancer Res..

[31] Marsischky G.T., Wilson B.A., Collier R.J. (1995). J. Biol. Chem..

[32] Thomas H.D., Calabrese C.R., Batey M.A., Canan S., Hostomsky Z., Kyle S., Maegley K.A., Newell D.R., Skalitzky D. (2007). Mol. Cancer Ther..

[33] Mason K.A., Buchholz T.A., Wang L., Milas Z.L., Milas L. (2014). Am. J. Clin. Oncol..

[34] Ekblad T., Schüler H., Macchiarulo A. (2013). FEBS J..

[35] Hilton J.F., Tran M.T., Shapiro G.I. (2013). Front. Biosci. (Landmark Ed)..

[36] Papeo G., Montagnoli A., Cirla A. (2013). Expert. Opin. Ther. Pat..

[37] Sonnenblick A., Azim H.A. Jr., Piccart M. (2015). Nat. Rev. Clin. Oncol..

[38] Curtin N.J., Szabo C. (2013). Mol. Aspects Med..

[39] De Lorenzo S.B., Hurley R.M., Kaufmann S.H. (2013). Front Oncol..

[40] Kuzminov A. (2001). Proc. Natl. Acad. Sci. USA..

[41] Chapman J.R., Boulton S.J. (2012). Mol. Cell Biol..

[42] Deindl S., Hota S.K., Blosser T.R., Prasad P., Bartholomew B., Zhuang X. (2013). Cell..

[43] Min I.M., Core L.J., Munroe R.J., Schimenti J., Lis J.T. (2011). Genes Dev..

[44] Patel A.G., Sarkaria J.N., Kaufmann S.H. (2011). Proc. Natl. Acad. Sci. USA..

[45] Bryant H.E., Thomas H.D., Parker K.M., Flower D., Lopez E., Kyle S., Meuth M., Curtin N.J., Helleday T. (2005). Nature.

[46] Farmer H., Lord C.J., Tutt A.N., Johnson D.A., Richardson T.B., Santarosa M., Dillon K.J., Hickson I., Knights C., Martin N.M. (2005). Nature.

[47] Donoho G., Brenneman M.A., Cui T.X., Donoviel D., Vogel H., Goodwin E.H., Chen D.J., Hasty P. (2003). Genes, Chromosomes Cancer..

[48] McCabe N., Turner N.C., Lord C.J., Kluzek K., Bialkowska A., Swift S., Giavara S., O’Connor M.J., Tutt A.N. (2006). Cancer Research.

[49] Li M., Yu X. (2013). Cancer Cell..

[50] Miwa M., Masutani M. (2007). Cancer Sci..

[51] Paddock M.N., Higdon R., Kolker E., Takeda S., Scharenberg A.M. (2011). DNA Repair..

[52] Fong P.C., Yap T.A., Tutt A., Wu P., Mergui-Roelvink M., Mortimer P., Swaisland H., Lau A., O’Connor M.J., Ashworth A. (2009). N. Engl. J. Med..

[53] Hutchinson L. (2010). Nat. Rev. Clin. Oncol..

[54] Chan S.L. (2010). Lancet..

[55] Bunting S.F., Callen E., Wong N., Chen H.T., Polato F., Gunn A., Bothmer A., Feldhahn N., Fernandez-Capetillo O., Cao L. (2010). Cell..

[56] Mukhopadhyay A., Elattar A., Cerbinskaite A., Wilkinson S.J., Drew Y., Kyle S., Los G., Hostomsky Z., Edmondson R.J., Curtin N.J. (2006). Clin. Cancer Res..

[57] Mendes-Pereira A.M., Brough R., McCarthy A., Taylor J.R., Kim J.S., Waldman T., Lord C.J., Ashworth A. (2009). EMBO Mol. Med..

[58] Buisson R., Coulombe Y., Launay H., Cai H., Stasiak A.Z., Stasiak A., Xia B., Masson J.Y. (2010). Nat. Struct. Mol. Biol..

[59] Williamson C.T., Turhan A.G., Zamò A., O’Connor M.J., Bebb D.G., Lees-Miller S.P. (2010). Mol. Cancer Ther..

[60] Villano J.L., Seery T.E., Bressler L.R. (2009). Cancer Chemother. Pharmacol..

[61] Boulton S., Pemberton L.C., Porteous J.K., Curtin N.J., Griffin R.J., Golding B.T., Durkacz B.W. (1995). Br. J. Cancer..

[62] Delaney C.A., Wang L.Z., Kyle S., Srinivasan S., White A.W., Calvert A.H., Curtin N.J., Durkacz B.W., Hostomsky Z., Maegley K. (2000). Clin. Cancer Res..

[63] Liu S.K., Coackley C., Krause M., Jalali F., Chan N., Bristow R.G. (2008). Radiother. Oncol..

[64] Liu X., Shi Y., Guan R., Donawho C., Luo Y., Palma J., Zhu G.D., Johnson E.F., Rodriguez L.E., Ghoreishi-Haack N. (2008). Mol. Cancer Res..

[65] Tentori L., Leonetti C., Scarsella M., d’Amati G., Portarena I., Zupi G., Bonmassar E., Graziaia G. (2002). Blood..

[66] Tentori L., Leonetti C., Scarsella M., D’Amati G., Vergati M., Portarena I., Xu W., Kalish V., Zupi G., Zhang J., Graziani G. (2003). Clin. Cancer Res..

[67] Palma J.P., Rodriguez L.E., Montgomery D., Ellis P.A., Bukofzer G., Niquette A., Liu X., Shi Y., Lasko L., Zhu G.D. (2009). Clin. Cancer Res..

[68] Donawho C.K., Luo Y., Penning T.D., Bauch J.L., Bouska J.J., Bontcheva-Diaz V.D., Cox B.F., DeWeese T.L., Dillehay L.E., Ferguson D.C. (2007). Clin. Cancer Res..

[69] Daniel R.A., Rozanska A.L., Mulligan E.A., Drew Y., Thomas H.D., Castelbuono D.J., Hostomsky Z., Plummer E.R., Tweddle D.A., Clifford S.C. (2010). Br. J. Cancer..

[70] Miknyoczki S.J., Jones-Bolin S., Prichard S. (2003). Mol. Cancer Ther..

[71] Calabrese C.R., Almassy R., Barton S., Batey M.A., Calvert A.H., Canan-Koch S., Durkacz B.W., Hostomsky Z., Kumpf R.A., Kyle S. (2004). J. Natl. Cancer Inst..

[72] Kaufmann S.H., Charron M., Burke P.J., Karp J.E. (1995). Cancer Research.

[73] El-Khamisy S.F., Masutani M., Suzuki H., Caldecott K.W. (2003). Nucleic Acids Research.

[74] Plo I., Liao Z.Y., Barceló J.M., Kohlhagen G., Caldecott K.W., Weinfeld M., Pommier Y. (2003). DNA Repair..

[75] Malanga M., Althaus F.R. (2005). Biochem. Cell Biol..

[76] Mattern M.R., Mong S.M., Bartus H.F., Mirabelli C.K., Crooke S.T., Johnson R.K. (1987). Cancer Research.

[77] Tentori L., Leonetti C., Scarsella M., Muzi A., Mazzon E., Vergati M., Forini O., Lapidus R., Xu W., Dorio A.S. (2006). FASEB J..

[78] Zander S.A., Kersbergen A., van der Burg E., de Water N., van Tellingen O., Gunnarsdottir S., Jaspers J.E., Pajic M., Nygren A.O., Jonkers J. (2010). Cancer Research.

[79] Ben-Hur E., Chen C.C., Elkind M.M. (1985). Cancer Research.

[80] Russo A.L., Kwon H.C., Burgan W.E., Carter D., Beam K., Weizheng X., Zhang J., Slusher B.S., Chakravarti A., Tofilon P.J., Camphausen K. (2009). Clin. Cancer Res..

[81] Saleh-Gohari N., Bryant H.E., Schultz N., Parker K.M., Cassel T.N., Helleday T. (2005). Mol. Cell. Biol..

[82] Barendsen G.W., van Bree C., Franken N.A.P. (2001). Int. J. Oncol..

[83] Leopold W.R., Sebolt-Leopold J.S. (1992). Chemical approaches to improved radiotherapy. Boston: Kluwer,.

[84] Albert J.M., Cao C., Kim K.W., Willey C.D., Geng L., Xiao D., Wang I.O., H. I.O., Sandler A., Johnson D.H., Colevas A.D. (2007). Clin. Cancer Res..

[85] Barreto-Andrade J.C., Efimova E.V., Mauceri H.J., Beckett M.A., Sutton H.G., Darga T.E., Vokes E.E., Posner M.C., Kron S.J., Weichselbaum R.R. (2011). Mol. Cancer Ther..

[86] Holl V., Coelho D., Weltin D., Hyun J.W., Dufour P., Bischoff P. (2000). Anticancer Res..

[87] Magan N., Isaacs R.J., Stowell K.M. (2012). Anticancer Drugs..

[88] Mason K.A., Valdecanas D., Hunter N.R., Milas L. (2008). Invest. New Drugs..

[89] Guggenheim E.R., Ondrus A.E., Movassaghi M., Lippard S.J. (2008). Bioorg. Med. Chem..

[90] Ohsaki K., Sakakibara S., Do E., Yada K., Yamanishi K. (2004). Virology Journal.

[91] Wang H., Tang Q., Maul G.G., Yuan Y. (2008). Virology Journal.

[92] Nakajima H., Ohkuma K., Ishikawa M., Hasegawa T. (2005). J. Pharmacol. Exp. Ther..

[93] Mandery K., Fromm M.F. (2012). Br. J. Pharmacol..

[94] Kopetz S. (2008). J. Clin. Oncol..

[95] Mahany J.J. (2008). J. Clin. Oncol..

[96] Kotova E., Tulin A.V. (2011). Meth. Mol. Biol..

[97] Maluchenko N.V., Kotova E., Chupyrkina A.A., Nikitin D.V., Kirpichnikov M.P., Studitsky V.M. (2015). Mol. Biol. (Mosc.)..

[98] Kotova E., Tulin A.V. (2010). Proc. Natl. Acad. Sci. USA..

[99] Dantzer F. (2013). FEBS J..

[100] O’Donnell A., Yang S.H., Sharrocks A.D. (2013). EMBO Rep..

[101] Thomas C. (2013). Mol. Aspects Med..

[102] Mouchiroud L., Auwerx J. (2013). Crit. Rev. Biochem. Mol. Biol..

[103] Bürkle A. (2013). Mol. Aspects Med..

[104] Ma Y., He X., Nie H., Hong Y., Sheng C., Wang Q., Xia W., Ying W. (2012). Curr. Drug Targets..

[105] Ying W. (2013). Scientifica (Cairo)..

[106] Baxter P., Xu Y., Swanson R.A. (2014). Transl. Stroke Res..

[107] Rosado M.M., Novelli F., Pioli C. (2013). Immunology..

[108] Nishikimi M., Kameshita I., Taniguchi T., Shizuta Y. (1982). J. Biol. Chem..

[109] Kameshita I., Taniguchi T., Shizuta Y. (1984). J. Biol. Chem..

